# SUMO-specific proteases/isopeptidases: SENPs and beyond

**DOI:** 10.1186/s13059-014-0422-2

**Published:** 2014-07-31

**Authors:** Arnab Nayak, Stefan Müller

**Affiliations:** Institute of Biochemistry II, Goethe University, Faculty of Medicine, Theodor-Stern-Kai 7, 60590 Frankfurt, Germany

**Keywords:** SUMO, SUMO isopeptidase, SUMO-specific protease, SENP, Desi-1, Desi-2, USPL1

## Abstract

We summarize the evolutionary relationship, structure and subcellular distribution of SUMO proteases (or SUMO isopeptidases). We also discuss their functions and allude to their involvement in human disease.

## Introduction

The ubiquitin-like SUMO (small ubiquitin-related modifier) system is a post-translational protein modification pathway in eukaryotes [1,2]. SUMOylation is a highly dynamic process, where deconjugation (deSUMOylation) is catalyzed by a family of cysteine proteases, termed SUMO-specific proteases or SUMO isopeptidases. A subset of these enzymes also functions in processing of the SUMO precursor proteins, which is a prerequisite for their conjugation. In this review, we summarize the current view of SUMO deconjugating enzymes. We discuss their evolutionary relationships, subcellular distributions and functions as well as their involvement in human disease.

SUMO belongs to the family of ubiquitin-like proteins. Like ubiquitin, it functions as a protein modifier that is covalently attached to ε-amino groups of lysine residues of target proteins [1,2]. Lower eukaryotes attach a single SUMO form (known as smt3) to target proteins, whereas the human SUMO system makes use of three related SUMO forms (SUMO1, SUMO2 and SUMO3). All SUMO/smt3 forms are expressed as precursor proteins that require carboxy-terminal proteolytic processing prior to their conjugation. Processing exposes a carboxy-terminal di-glycine motif, which is essential for conjugation. Subsequent conjugation proceeds by a pathway that typically involves an E1-E2-E3 enzymatic cascade. Modification by SUMO generally controls protein-protein interactions by recruiting binding partners that harbor specific SUMO-interaction motifs (SIMs).

Regulated deconjugation of SUMO from its substrates is a central element of the SUMO system as it assures the plasticity of protein interaction networks. The deconjugation process is catalyzed by a family of cysteine proteases, termed SUMO isopeptidases or SUMO-specific proteases. Members of this enzyme class function as deconjugating enzymes for isopeptide-linked SUMO-protein conjugates and also depolymerize isopeptide-linked poly-SUMO2/3 chains. Moreover, some family members act as processing factors for the carboxy-terminal maturation of the SUMO precursor. The known SUMO-specific isopeptidases and proteases are cysteine proteases that are classified into three distinct families: the Ulp/SENP (ubiquitin-like protease/sentrin-specific protease) family, the Desi (deSUMOylating isopeptidase) family and USPL1 (ubiquitin-specific peptidase-like protein 1).

## Gene organization and evolutionary history

All identified SUMO isopeptidases/proteases are cysteine proteases. They share a similar catalytic mechanism but belong to different superfamilies that are distinguished by the fold of their respective catalytic domains.

**Figure 1:**
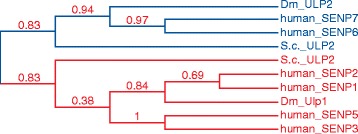
**Evolutionary relationship of Ulp/SENP family members.** The phylogenetic tree displays the relationship between *Saccharomyces cerevisiae* (S.c*.*), *Drosophila melanogaster* (Dm) and human Ulp/SENP family members. Confidence numbers generated by the bootstrapping procedure are shown for each branch in the tree. The following sequences were used for input: SENP1 UniProtKB, Q9P0U3; SENP2 UniProtKB, Q9HC62; SENP3 UniProtKB, Q9H4L4; SENP5 UniProtKB, Q96HI0; SENP6 UniProtKB, Q9GZR1; SENP7 UniProtKB, Q9BQF6; Dm_ULP1, GenBank AAF48933.1; Dm_ULP2 (Velo; verloren), GenBank AAS65070.1; S.c._ULP1, UniProtKB/Swiss-Prot Q02724.1; S.c._ULP2, UniProtKB/Swiss-Prot P40537.

Ulp/SENP proteins belong to the C48 family subgroup of the CE superfamily of thiol proteases whose founder member is Ulp1, first discovered in the yeast *Saccharomyces cerevisiae* [3]. Subsequently, Ulp2 was identified as a second SUMO-deconjugating enzyme in yeast [4]. Ulp1/2-related isopeptidase genes are also found in the genome of *Drosophila melanogaster* [5,6]. In higher eukaryotes, the family is more diverse. Seven human proteins were initially classified as Ulp family members and annotated as SENPs [7]. (The term sentrin was coined by Ed Yeh as an alternative name for SUMOs.) Notably, however, later experimental work revealed that SENP8 acts on the ubiquitin-family member Nedd8, but not on SUMO paralogs [8,9]. The human genome therefore encodes six dedicated SUMO-specific members of the Ulp/SENP family: SENP1, SENP2, SENP3, SENP5, SENP6 and SENP7. Sequence analyses and phylogenetic comparison of the human SENPs with Ulp1 and Ulp2 from lower eukaryotes illustrate that SENP1, SENP2, SENP3 and SENP5 are evolutionarily related to the Ulp1 branch, whereas SENP6 and SENP7 are related to the Ulp2 branch (Figure 1). The tree further reveals the pairwise similarity of SENP1 to SENP2, SENP3 to SENP5 and SENP6 to SENP7.

The deSUMOylating isopeptidases Desi-1 and Desi-2 belong to the evolutionarily distinct C97 family of cysteine proteases [10]. Orthologs of Desi-1 and Desi-2 are found in plants and metazoa, but are missing in lower eukaryotes such as yeast.

USPL1 is, to date, the only known mammalian SUMO-specific protease of the C98 family [11]. USPL1-related deconjugases are found in metazoan vertebrates and invertebrates. USPL1 is not related to the Ulp/SENP and Desi families, but the catalytic domain of USPL1 shows homology to the C19 family of ubiquitin-specific proteases. For example, within this region, USPL1 shares around 20% sequence identity with the ubiquitin-deconjugating enzyme USP1.

## Characteristic structural features

**Figure 2:**
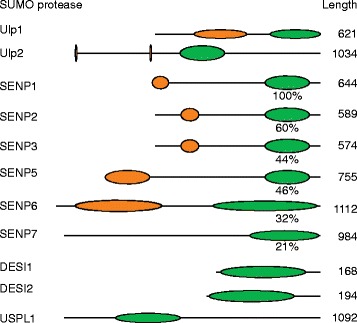
**Structural organization of SUMO-specific-proteases/isopeptidases.** The domain organizations of Ulp/SENPs and Desi family members are shown. Green ovals represent the catalytic domain. The sequence determinants that are responsible for subcellular targeting are represented by orange ovals. The length of the proteins as total number of amino acids is presented on the right side. For the catalytic domains of SENP family members, sequence identity shared with SENP1 is also shown.

The common characteristic of the Ulp/SENP family is their conserved catalytic domain, which spans around 200 amino acids in the carboxy-terminal part of the protein (Figure 2). This domain has sequence and structural similarity to the catalytic region of adenoviral proteases, which cleave viral and cellular proteins. The human Ulp/SENP family members share 20 to 60% sequence identity within their catalytic domains. The SENP1-SENP2, SENP3-SENP5 and SENP6-SENP7 pairs show the highest degree of similarity to each other. SENP6 and SENP7 are most divergent from the rest of the family and harbor conserved sequence insertions within their catalytic domains. Structural data describing the catalytic domain of yeast Ulp1 and human SENP1 and SENP2 in complex with SUMO precursors or isopeptide-linked SUMO-RanGAP1 conjugates revealed important insights into the catalytic features of this enzyme class [12-16]. The active site cysteine residue is embedded in a typical catalytic triad (Cys-His-Asp). An additional invariant Gln residue in close proximity stabilizes the transition state during catalysis. The substrate accesses the catalytic site through a shallow tunnel, in which conserved Trp residues are essential for positioning the di-glycine motif and the scissile bond over the active site. A remarkable finding was that the scissile bond of the pre-SUMO variants or a SUMO-conjugate is oriented in a *cis* configuration for cleavage [14,15].

In addition to their conserved catalytic domain, all SENPs have amino-terminal regions of variable length that have crucial regulatory functions. These regions frequently contain interaction domains for cellular adaptor proteins that determine the subcellular distribution of Ulp/SENP family members [17-19]. Post-translational modifications, such as phosphorylation or ubiquitylation, within these regions provide additional regulatory layers for the recruitment of binding partners or the control of Ulp/SENP stability [20-23]. Interestingly, most SENPs contain one or more SIM modules in their amino-terminal region, which probably contribute to the selection of substrates or facilitate the targeting of specific SENPs to poly-SUMO chains.

Desi-1 and Desi-2 are small proteins characterized by PPPDE (permutated papain fold peptidases of the double-stranded RNA viruses and eukaryotes) domains of around 140 amino acids. Desi-1 and Desi-2 share about 20% sequence identity within this region. Structural data from Desi-1 revealed that the protein forms a homodimer, in which the groove between the two subunits forms the active site. This region contains two conserved cysteine and histidine residues that form a catalytic dyad [10,24]. Interestingly, the active-site groove of Desi-1 is occupied by its own carboxy-terminal segment [24], which is very different from the open cleft in SENPs.

The catalytic domain of USPL1 contains a catalytic triad composed of Cys-His-Asp-residues [11,25].

## Localization

**Figure 3:**
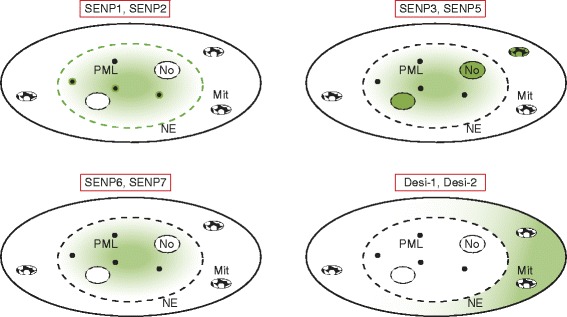
**Schematic representation of the subcellular distribution of mammalian SUMO-specific isopeptidases of the SENP and Desi families.** The predominant subcellular distribution of the respective SENPs is indicated by the green color. Mit, mitochondria; NE, nuclear envelope; No, nucleolus; PML, promyelocytic leukemia nuclear bodies.

The different SUMO isopeptidases have characteristic subcellular distributions, which seems to be a way of restricting their activity to a specific set of substrates. The Ulp/SENP family members are predominantly concentrated in distinct subnuclear regions (Figure 3). SENP1 and SENP2, as well as the yeast Ulp1 enzyme, are concentrated at the nuclear envelope through their interaction with components of the nuclear pore complex [18,26-28]. Within the nucleus SENP1 and SENP2 are excluded from the nucleolus, but enriched in nuclear foci that partially overlap with PML nuclear bodies. In mitosis, SENP1 and SENP2 redistribute from the nuclear envelope to the kinetochore [29]. It is worth noting that despite its predominately nuclear localization, SENP2 was reported to shuttle between the nucleus and the cytoplasm [30]. Moreover, distinct splice variants of SENP2 may exhibit specific subcellular distributions [31].

SENP3 and SENP5 are compartmentalized in the nucleolus, where they act on proteins that are involved in the early steps of ribosome maturation [17,32-34]. A subfraction of SENP3 and SENP5 also resides in the nucleoplasm and the cytoplasm. Interestingly, at G_2_/M transition, prior to nuclear envelope breakdown, SENP5 translocates to the mitochondrial surface [35]. SENP6 and SENP7 mainly exhibit a nucleoplasmic distribution. In contrast to SENPs, Desi family members are primarily concentrated in the cytoplasm [35]. USPL1 is a predominantly nuclear protein and co-localizes with coilin in Cajal bodies [11,25].

## Function

### SUMO maturation versus SUMO deconjugation

**Figure 4:**
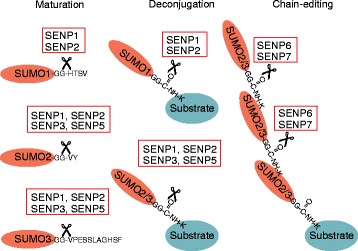
**SUMO processing and SUMO deconjugation activities of human SENP family members.** Schematic representation of SENP processing and deconjugation activities towards distinct SUMO paralogs. The left part summarizes the activities of SENPs in maturation/processing of human SUMO paralogs. The maturation process removes the amino acids carboxy-terminal to a di-glycine (GG) motif (sequences are given for the human SUMO variants). The middle part describes the specificity for deconjugating distinct SUMO forms from substrates (isopeptidase activity). The right part highlights the activity of SENP6 and SENP7 for editing lysine (K)-linked SUMO2/3 chains. These chains are predominantly formed by SUMO2 and SUMO3 and are preferentially linked via K11. The figure tries to integrate data from biochemical *in vitro* experiments and cell-based assays.

Ulp/SENPs can act as deconjugating or maturation/processing enzymes. The yeast Ulp family was covered in a recent review [31] and will not be further discussed here. Distinct human SENPs exert different activities in either processing or deconjugation (Figure 4). Moreover, preferences for specific SUMO paralogs have been reported for specific SENPs as outlined below. The maturation process removes the amino acids carboxy-terminal to a di-glycine motif, whose exposure is essential for conjugation. The substrate specificity of SENPs for processing is determined by the amino acids that are found carboxy-terminal to the di-glycine motif.

Biochemical experiments have established a role for SENP1 in both SUMO deconjugation and SUMO processing. The data show that SUMO-1 is processed most efficiently, followed by SUMO-2 and SUMO-3. By contrast, at least *in vitro*, SENP1 displayed little specificity in its ability to deconjugate the different SUMO paralogs from SUMO-modified substrates [13,15,16]. Recent genetic data from mice, however, suggest that *in vivo* SENP1 has only a limited role in SUMO-2 and SUMO-3 deconjugation, but is essential for deSUMOylating SUMO1-modified proteins [36]. Biochemical experiments demonstrate that SENP2 catalyzes SUMO deconjugation more efficiently than SUMO processing and is most efficient for SUMO2 over SUMO1 and SUMO3 [14,37,38]. The SENP3-SENP5 couple shows a very strong preference for processing and deconjugation of SUMO2 and SUMO3 and does not exhibit significant processing and deconjugase activity for SUMO1 [32,39]. SENP6 and SENP7 exert only very weak processing activity and are also rather inefficient in deconjugating monomeric SUMO from substrates. Importantly, however, SENP6 and SENP7, as well as the related Ulp2 from yeast, are excellent enzymes for deconjugating SUMO moieties from SUMO2-SUMO3 di-SUMOylated substrates and from polymeric chains of SUMO2 and SUMO3 [39-42]. Their main function seems to be the editing of lysine-linked SUMO-SUMO chains.

In contrast to Ulp/SENP family members, the Desi-1 and Desi-2 enzymes exert isopeptidase activity on a selected substrate, but have only an extremely low processing activity for the pre-SUMO precursor protein [10,24]. USPL1 is active in processing and deconjugation.

### Substrate specificity of SUMO proteases

With only nine SUMO isopeptidases identified to date, the SUMO-deconjugating machinery appears to be less complex than the de-ubiquitylating system. Even if there are unidentified SUMO isopeptidases or additional alternative splice variants of SENPs, their total number is likely to remain far below the currently known 100 ubiquitin deconjugases. The situation is similar when comparing the number of E3 ubiquitin ligases and E3 SUMO ligases. Why the modification and demodification of hundreds of different SUMO conjugates are controlled by only a limiting set of enzymes is a central question in the field. One scenario is that the SUMO system, including the deconjugation machinery, coordinately regulates groups of proteins that are functionally and physically linked [43]. In this scenario, a given SUMO isopeptidase is likely to act on a larger set of proteins, which in many cases are associated in larger protein complexes. For example, the human SENP3 enzyme deconjugates a number of SUMO2/3 conjugates at nucleolar pre-60S ribosomes [33,34,44-47]. The concept of group modification or demodification complicates the interpretation of experimental approaches that had concentrated on individual SUMO substrates for a given isopeptidase. In the following, we will try to integrate the available data to provide a more general picture and to define cellular pathways that are controlled by a distinct SUMO isopeptidase.

### Cellular pathways regulated by SUMO-specific isopeptidases

#### SENP1

The inactivation of transcription factors by SUMO conjugation and their activation by SUMO deconjugation is a recurrent theme. SENP1-mediated deSUMOylation seems to be crucial for the activation of transcriptional programs in innate immune responses and in the development of B and T cells. In these processes, IRF8, STAT5 and Bcl11b have been defined as relevant SENP1 targets [48-50], but SENP1 probably acts on a broader spectrum of transcription factors in these pathways and other processes. Along this line, it has been shown that deSUMOylation of HIF1α by SENP1 under conditions of hypoxia is required for stabilization of HIF1α and the expression of HIF1α target genes. Recruitment of SENP1 to specific substrates could be coordinated by post-translational modifications as exemplified by the phospho-dependent binding of SENP1 to Bcl11b [50].

In mitotic cells, SENP1 appears to target selected substrates at the kinetochore. This is critical for mitotic progression because the knockdown of SENP1 delays sister chromatid separation at metaphase [29].

#### SENP2

Like SENP1, SENP2 is involved in the regulation of gene expression programs in developmental processes. Yeh and co-workers [51] demonstrated that deletion of the *SENP2* gene in mice causes defects in cardiac development resulting from the reduced expression of Gata4 and Gata6. This reduced expression has been linked to the lack of SENP2-mediated deSUMOylation of a subunit of the polycomb repressive complex 1 (PRC1). PRC1 represses transcription of Gata4, Gata6 and numerous other developmental regulator genes, indicating a central role for SENP2 in early embryonic development.

#### SENP3

SENP3 has a well-established function in the control of ribosome biogenesis and particularly affects the maturation of the 28S rRNA [33,34]. This is likely to involve the SENP3-catalyzed removal of SUMO2 or SUMO3 from various 60S maturation factors acting on nucleolar pre-60S ribosomal particles. In addition to its role in ribosome biogenesis, a nucleoplasmic subfraction of SENP3 controls transcriptional processes through demodification of transcriptional co-regulators and components of chromatin-modifying complexes [52]. Recent data show that SENP3-mediated deSUMOylation controls the expression of osteogenic differentiation factors and of other developmental regulators through deSUMOylation of MLL1/2 histone-methyltransferase complexes [53].

SENP3 abundance and subcellular distribution are tightly regulated by environmental stimuli. Redox-sensitive cysteine residues in the amino-terminal region trigger the stabilization of SENP3 in response to redox stress, whereas the PERK1 kinase pathway induces its lysosomal degradation in response to oxygen or glucose deprivation [20,54]. In mitosis, SENP3 is heavily phosphorylated, which may control its substrate specificity and/or subcellular distribution [21].

#### SENP5

The nucleolar function of SENP5 is also related to the ribosome biogenesis pathway, where it is involved in RNA polymerase I-mediated transcription of the 47S rRNA [34]. As mentioned above, upon G_2_/M transition, SENP5 translocates to mitochondria, where the deSUMOylation of mitochondrial proteins seems to drive mitochondrial fragmentation during mitosis [35,55].

#### SENP6

SENP6 is the principal chain-editing enzyme in human cells and accordingly regulates multiple signaling pathways that are controlled by poly-SUMOylation. A well-characterized poly-SUMO2/3-regulated process is SUMO-mediated ubiquitylation by SUMO-targeted ubiquitin-ligases (StUbLs). StUBLs, whose prototypic member is RNF4, are poly-SUMO2/3-binding ubiquitin ligases that are recruited to poly-SUMOylated substrates to trigger their ubiquitylation. Among the established targets of RNF4 are the promyelocytic leukemia (PML) protein and the inner kinetochore protein CENP-I. SENP6 counterbalances this poly-SUMO-RNF4-dependent degradation pathway by preventing the polySUMOylation of PML or CENP-I, as evidenced by the accumulation of both proteins in the absence of SENP6 [56-58]. Other defined substrates for SENP6 are the NF-κB regulator NEMO and the replication factor RPA70 [59,60]. How SENP6 is selectively targeted to its substrates under specific conditions is currently not understood.

#### SENP7

Similar to SENP6, SENP7 preferentially acts on SUMO-SUMO chains. Small interfering RNA (siRNA)-mediated SENP7 depletion experiments point to the crucial involvement of SENP7 in chromatin remodeling and chromatin dynamics. It has been proposed that chromatin relaxation in response to DNA damage is promoted by SENP7-mediated removal of SUMO2/3 chains from the KRAB-associated protein 1 (KAP1) [19]. This allows the recruitment of the chromatin remodeler CHD3, which triggers chromatin relaxation. Importantly, the heterochromatin protein HP1 seems to function as the chromatin-targeting adaptor for SENP7 and may itself be a substrate for SENP7. Interestingly, recent work identified a shorter splice variant of SENP7, which lacks the HP1 binding domain and accordingly is unable to deSUMOylate HP1 [61]. These data exemplify the possible role of alternative splicing of SENPs for substrate selection.

#### Desi-1/2 and USPL1

Only limited functional data on Desi-1/2 and USPL1 are currently available. Desi-1 and -2 seem to have a more restricted substrate specificity, with the transcriptional repressor BZEL being the only substrate identified to date [10]. Consistent with its localization to Cajal bodies, USPL1 has been shown to be important for small nuclear ribonucleic particle (snRNP) assembly and pre-mRNA splicing, but the relevant substrates have not yet been identified [11,25].

## Frontiers

Among the most burning questions in the field is the target specificity of distinct SUMO isopeptidases. Quantitative mass-spectrometry approaches that monitor SUMOylation in cells or tissues in which specific family members are depleted might be one promising approach to answer this question. Unraveling the substrate specificity of distinct SENP splice variants would also be an important aspect along this line. Considering that USPL1 exerts essential non-catalytic functions [11], it is also crucial to define potential functions of other SUMO isopeptidases that are not linked to their catalytic activity. Knock-in mice expressing catalytic-dead variants of the respective enzymes would be the best model system to tackle this search.

Future work will also need to uncover how the misregulation of SENPs is linked to human disease. Most studies have focused on an involvement of SENPs in the development and/or progression of cancer [62]. Overexpression of SENP1 has been correlated with prostate cancer aggressiveness and metastatic potential [63-65]. This is at least partially mediated through induction of HIF1α-dependent signaling pathways. SENP1 additionally activates other oncogenic signaling pathways, such as c-Jun and androgen-receptor-mediated transcription. Like SENP1, SENP3 accumulates in several human cancers, with colon carcinomas having the highest ratio of SENP3 expression [54,66]. These and other data indicate a potential significance for SENPs as diagnostic markers and also make this enzyme class an attractive drug target in distinct human tumors [67,68].

## Abbreviations

Desi: deSUMOylating isopeptidase
KAP1: KRAB-associated protein 1
PML: promyelocytic leukemia
siRNA: small interfering RNA
snRNP: small nuclear ribonucleic particle
StUbL: SUMO-targeted ubiquitin-ligase
SUMO: small ubiquitin-related modifier
Ulp/SENP: ubiquitin-like protease/sentrin-specific protease
USPL1: ubiquitin-specific peptidase-like protein 1
